# Alteration of Time Intervals in Patients With Hypertrophic Cardiomyopathy During an Exercise Echocardiography

**DOI:** 10.4021/cr35w

**Published:** 2011-03-25

**Authors:** Angela Zagatina, Nadezhda Zhuravskaya, Tatyana V. Tyurina

**Affiliations:** a Leningrad Regional Cardiologic Dispensary, St. Petersburg, Russian Federation

**Keywords:** Hypertrophic cardiomyopathy, Diastolic function, Stress echocardiography

## Abstract

**Background:**

Hypertrophic cardiomyopathy (HCM) is often associated with diastolic dysfunction. Theoretically, a more marked alteration of diastolic function is revealed during exercise.

**Methods and Results:**

We studied 84 persons: 1) 25 patients with HCM, 2) 25 patients with essential arterial hypertension (AH) and 3) 34 healthy controls. Each person performed a treadmill echocardiography. Before and after work, the following parameters were measured: the time interval between the QRS complex and the onset of mitral early diastolic filling velocity (TE), the interval between the QRS complex and the onset of peak early tissue mitral annular velocity (Te’), the isovolumetric relaxation time over the difference of TE and Te’ ratio (IVRT/(TE-Te’)), and changes of the time parameters during the stress test. In comparison with hypertensive and control groups, HCM patients at rest showed a significantly longer TE (448 ± 55 vs. 423 ± 33 vs. 417 ± 24 ms, P < 0.04) and Te’ (446 ± 48 vs. 403 ± 44 vs. 416 ± 38 ms, P < 0.003). After stress the HCM group had a longer Te’ (355 ± 59 vs. 299 ± 40 vs. 292 ± 30 ms, P < 0.000004) and a higher IVRT/(TE-Te’) ratio (3.1 ± 1.5 vs. 0.9 ± 2.4 vs. 1.7 ± 1.2, P < 0.002).

**Conclusions:**

HCM patients show an alteration in the time parameters not only compared to healthy persons but to hypertensive patients as well.

## Introduction

Hypertrophic cardiomyopathy is a genetic disease characterized by left ventricular hypertrophy and myocyte disarray [1-3]. This results in a delay in ventricular relaxation, an increase in left ventricle stiffness and a gradual elevation in filling pressure. Delayed relaxation is manifested by a prolonged isovolumetric relaxation time (IVRT) [4, 5]. With increasing left ventricular end-diastolic and filling pressures, IVRT becomes shorter, because there is an earlier equalization between left ventricle and left atrium pressures in diastole [4]. However, the time to reach peak early mitral annular velocity remains long. The time interval between the onset of early mitral diastolic filling velocity and the onset of early mitral annular velocity is strongly dependent on the time constant for left ventricle relaxation [6, 7].

Theoretically, patients with HCM, having diastolic dysfunction, should exhibit a difference in these time intervals in comparison with healthy persons. It should be more pronounced during exercise, because there would be a more marked alteration in diastolic function at those times.

The aim of the study was to investigate the alteration in the time intervals in HCM during exercise tests in comparison with healthy persons and to reveal the particulars of these alterations in comparison with patients having arterial hypertension.

## Methods

### Subjects

We studied 84 persons (3 groups): 1) 25 patients with HCM without any systolic obstructions in the left ventricular outflow tract at rest. The diagnostic criteria for HCM was a maximal left ventricular wall thickness greater than or equal to 15 mm in the absence of other cardiac or systemic disease [8]. 2) Twenty-five patients with arterial hypertension (comparable in their age and sex ratios). 3) Thirty-four healthy persons (comparable in their age and sex ratios).

Participants were excluded if they were not in sinus rhythm or if they had a history of coronary artery disease, regional wall abnormalities before or after the stress tests, valve disease, pericardial disease or cor pulmonale.

### Conventional echocardiography

M-mode, 2D and Doppler standard echocardiographic examinations were performed in all subjects at rest, as proposed by respective guidelines [9, 10] using Sonoline G 60 S (Simens, Japan).

In the process the left atrial volume was measured using Simpson’s rule. The left ventricular mass was estimated based on an area-length formula. For assessment and grading diastolic dysfunction we also measured and calculated transmitral flow parameters including the early (E) and late (A) diastolic filling velocities, the E/A ratio, the E deceleration time (DT), and the A wave duration (Adur); isovolumetric relaxation time (IVRT); the ratio of the systolic (S) and diastolic (D) velocities (S/D), the late diastolic A wave (A’) and duration of A’ (A’dur) into the left atrium; the peak early diastolic lateral mitral annular velocities (e’); E/e’ ratio; the difference in the pulmonary venous and the transmitral A wave durations (A’-Adur).

### Treadmill test

Heart rate, blood pressure and a 12-lead ECG were obtained at baseline and at 3-min stages. Patients were encouraged to perform a treadmill exercise test (Bruce protocol, modified Bruce) until exhaustion or until they reached an endpoint. Endpoints were standard for exercise tests [11].

### Stress echocardiography

**Figure 1:**
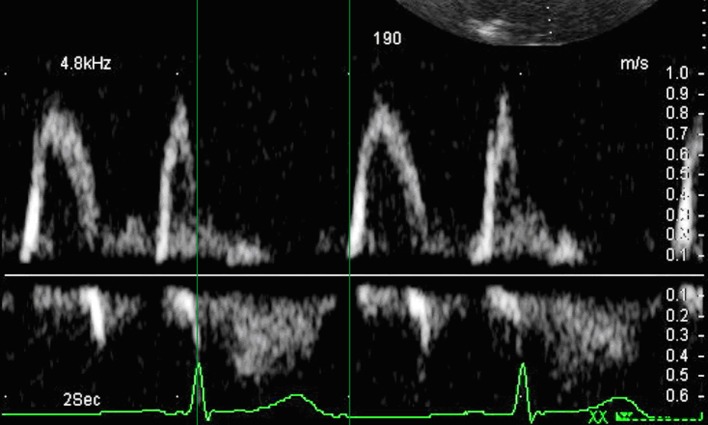
Time interval between the QRS complex and the onset of mitral early diastolic filling velocity - TE.

**Figure 2:**
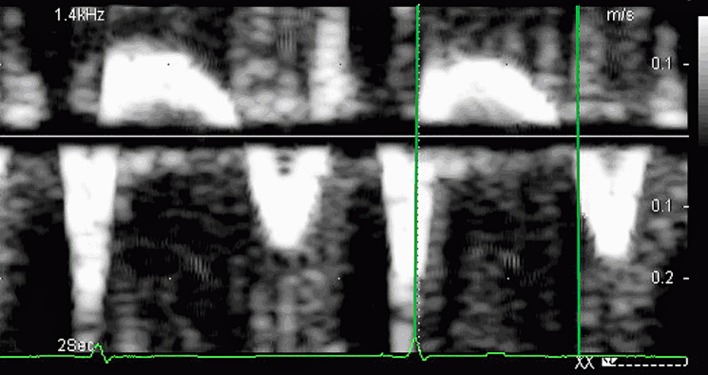
The interval between the QRS complex and the onset of peak early tissue mitral annular velocity - Te’.

Before and after maximal tolerated workloads the following parameters were measured and calculated: the time interval between the QRS complex and the onset of mitral early diastolic filling velocity (TE) (Fig. 1), the interval between the QRS complex and the onset of peak early tissue mitral annular velocity (Te’) (Fig. 2), the isovolumetric relaxation time over the difference between the TE and Te’ (IVRT/(TE-Te’)), the change of post-stress and rest TE (ΔTE), and the change of post-stress and rest Te’ (ΔTe’). All the post-stress images were acquired till 5th minutes after stress. Image acquisition was performed on-line and stored for off-line analysis. All patients performed without beta-blockers.

### Statistical analysis

Continuous variables were described by means and standard deviations (STATISTICA version 6). For multiple comparisons ANOVA was performed. Mann-Whitney U Test was used for comparison of ratios. Pearson Chi-square was used for crosstabulation tables. Wilcoxon matched pairs test was used for comparing changes in the time ratios during exercise. Significance was determined as P < 0.05.

## Results

### General data and rest echocardiography

Eleven of the HCM patients (44%) had a history of dyspnea. General and echocardiographic parameters for diastolic function are summarized in Table 1. All the patients had normal systolic function before and after the stress tests. Despite the significant abnormality of the left ventricular mass index, and left atrial volume index, only four patients (16%) with HCM at rest had a classified type of diastolic dysfunction according to “Recommendations for the Evaluation of Left Ventricular Diastolic Function” [12]. The majority of HCM patients (75 - 85%) had E/A, DT, A’-Adur and S/D values within normal range. At the same time IVRT was longer in 56% of HCM patients. The ratio of the mitral early diastolic to the annular early diastolic velocities (E/e’) was less than 8 in 22 persons with HCM (88%), and from 8 to 15 in 3 patients. One of the latter had no other signs of abnormal filling and diastolic dysfunction.

**Table 1:** General and Echocardiographic Parameters

	HCM group	AH group	Normal controls	P-value 1	P-value 2
	N = 25	N = 25	N = 34		
General data					
Age (years)	40 ± 13	46 ± 7	45 ± 7	NS	NS
Gender (M/F)	12/13	17/8	20/12	NS	NS
BSA (m^2^)	1.83 ± 0.20	2.00 ± 0.21	1.85 ± 0.20	0.006	0.003
Echocardiographic data					
IMM (g/m^2^)	149 ± 44	86 ± 24	71 ± 15	0.0000001	0.008
ILAVol (ml/m^2^)	35 ± 12	29 ± 8	28 ± 7	0.04	NS
IVRT (ms)	101 ± 24	96 ± 18	80 ± 16	NS	0.0003
E/A	1.8 ± 0.8	1.2 ± 0.3	1.4 ± 0.4	0.0003	0.03
DT (ms)	198 ± 53	210 ± 56	182 ± 36	NS	0.005
A duration (ms)	113 ± 24	142 ± 26	133 ± 30	0.0003	NS
PV S/D	1.3 ± 0.4	1.3 ± 0.3	1.1 ± 0.3	NS	0.007
PV A’ (cm/s)	0.32 ± 0.07	0.30 ± 0.03	0.30 ± 0.05	NS	NS
A’ duration (ms)	115 ± 34	103 ± 27	93 ± 17	NS	NS
E/e’	6.5 ± 2.0	4.7 ± 1.5	4.1 ± 0.8	0.0008	0.05

HCM: hypertrophic cardiomyopathy; AH: arterial hypertension; BSA: body surface area; IMM: index myocardial mass; ILAVol: index of the left atrial volume; IVRT: isovolumetric relaxation time; E/A: ratio of the early filling (E) to the late diastolic filling (A) velocities; DT: deceleration time of the early diastolic velocity; PV S/D: ratio of the systolic (S) to early diastolic (D) velocities in the pulmonary vein; PV A’: the late diastolic velocity in the pulmonary vein; E/e’: ratio of the mitral to the annular early diastolic velocities; NS: non-significant difference.

P-value 1 was defined for comparison between HCM and AH groups.

P-value 2 was defined for comparison between AH and control groups.

The patients in the AH group had histories of high blood pressure for 6.7 ± 5.7 years (more than 5 years in 16 patients, 64%). Like the HCM groups at rest, the majority of AH patients (18 subjects, 72%) had no signs of diastolic dysfunction. Five of them (20%) had impaired relaxation patterns and two patients (8%) had pseudonormal types of diastolic dysfunction. The AH group was comparable with the HCM group in their age and sex ratios so these patients were younger-aged with a lesser degree of arterial hypertension in comparison with the general population. In spite of that, these patients had significant differences in their BSA, IMM, TD, IVRT, S/D and E/e’ than controls (Table 1).

### Medical treatment

Eleven of the HCM patients (44%) were being treated with beta-blockers. Sixteen patients in the AH group (64%) were being given beta-blockers, nine subjects (36%) - ACE inhibitors, and 2 patients were additionally treated with thiazide diuretics.

All the medication had been stopped prior to the test.

### Treadmill test

The reasons for terminating exercise are summarized in Table 2.

**Table 2:** Exercise End Points

	HCM group	AH group	Normal controls
	N = 25	N = 25	N = 34
Submaximal heart rate	52%	80%	97%
Fatigue	16%	8%	3%
Hypertensive response	0%	4%	0%
Dyspnea	12%	6%	0%
Drop in systolic blood pressure	8%	0%	0%
Chest pain	8%	0%	0%

HCM: hypertrophic cardiomyopathy; AH: arterial hypertension

The maximum heart rate of HCM patients was 147 ± 21 bpm, the maximum heart rate of patients with arterial hypertension was 153 ± 13 bpm (P = 0.18). There were significantly higher heart rates in control persons (158 ± 8 bpm, P < 0.009). Maximum workload achieved on the treadmill was 10.5 ± 4.2 MET for HCM patients, 11.3 ± 3.1 MET for patients with arterial hypertension (P = 0.42) and 12.0 ± 4.0 MET for controls (P = 0.18).

After exercise, seven of the HCM persons (28%) had a significant obstruction in the left ventricular outflow tract.

The echocardiographic parameters of diastolic function immediately after exercise are summarized in Table 3.

**Table 3:** Echocardiographic Parameters of Diastolic Function After the Stress

	HCM group	AH group	Normal controls	P-value
	N = 25	N = 25	N = 34	
IVRT (ms)	80 ± 20	82 ± 14	66 ± 12	NS
E/A	1.7 ± 0.6	1.1 ± 0.3	1.3 ± 0.3	0.0003
DT (ms)	174 ± 44	184 ± 54	175 ± 32	NS
A duration (ms)	118 ± 25	127 ± 29	134 ± 29	NS
PV S/D	1.2 ± 0.5	1.3 ± 0.3	1.2 ± 0.2	NS
PV A’ (cm/s)	0.34 ± 0.08	0.34 ± 0.04	0.34 ± 0.06	NS
A’ duration (ms)	110 ± 24	100 ± 15	93 ± 17	NS
E/e’	5.9 ± 2.0	4.3 ± 1.4	3.9 ± 0.9	0.007

HCM: hypertrophic cardiomyopathy; AH: arterial hypertension; IVRT: isovolumetric relaxation time; E/A: ratio of the early filling (E) to the late diastolic filling (A) velocities; DT: deceleration time of the early diastolic velocity; PV S/D: ratio of the systolic (S) to early diastolic (D) velocities in the pulmonary vein; PV A’: the late diastolic velocity in the pulmonary vein; E/e’: ratio of the mitral to the annular early diastolic velocities; NS: non-significant difference.

P-value was defined for comparison between HCM and AH groups.

### Time parameters

In comparison with hypertensive and control groups, HCM patients at rest showed a significantly longer TE (448 ± 55 vs. 423 ± 33 vs. 417 ± 24 ms, P < 0.04) and Te’ (446 ± 48 vs. 403 ± 44 vs. 416 ± 38 ms, P < 0.003). After stress the HCM group had a longer Te’ (355 ± 59 vs. 299 ± 40 vs. 292 ± 30 ms, P < 0.000004) and a higher IVRT/(TE-Te’) ratio (3.1 ± 1.5 vs. 0.9 ± 2.4 vs. 1.7 ± 1.2, P < 0.002). The time parameters for patients with hypertension did not significantly differ from controls before and after the exercise test.

There was not a significant difference between the peak heart rates of HCM and AH groups. However, all the parameters were obtained after stress during 5 minutes when the velocity of heart rate recovery was different in different patients. In addition the control group had a higher peak heart rate. Taking into account that the time parameters are significantly dependent on heart rate, we assessed the relation of these parameters to R-R duration in the acquired cycle.

The obtained data showed time TE (until mitral valve opening) to the overall time of R-R interval, and time Te’ (until the onset of diastolic wall motion relaxation) to the overall time of R-R interval (Table 4).

$$\frac{{\text{T}}_{\text{E}}}{\text{R}-\text{R}}\;\frac{{\text{T}}_{\text{e}\prime }}{\text{R}-\text{R}}\;$$

**Table 4:** The Relative Time Parameters

	HCM group	AH group	Normal controls	P-value
	N = 25	N = 25	N = 34	
TE/R-R rest	0.49 ± 0.07	0.51 ± 0.07	0.50 ± 0.05	NS
Te’/R-R rest	0.48 ± 0.07	0.48 ± 0.06	0.50 ± 0.05	NS
TE/R-R post stress	0.52 ± 0.05	0.53 ± 0.06	0.53 ± 0.04	NS
Te’/R-R post stress	0.54 ± 0.07	0.49 ± 0.06	0.49 ± 0.06	0.03

HCM: hypertrophic cardiomyopathy; AH: arterial hypertension; TE/R-R: ratio, the time interval between the QRS complex and the onset of mitral early diastolic filling velocity/the time of R-R interval; Te’/R-R: ratio, the interval between the QRS complex and the onset of peak early tissue mitral annular velocity/the time of R-R interval; NS: non-significant difference.

The AH and control groups had the same relative time until the onset of diastolic wall motion relaxation before and after the stress. That is Te’/R-R was the same before and immediately after the exercise in these groups. Whereas, this ratio (Te’/R-R) increased significantly after the exercise in HCM patients (0.48 ± 0.07 vs. 0.54 ± 0.07, P < 0.0004).

## Discussion

Diastolic dysfunction is important to assess for many reasons. There are numerous studies that have established the prognostic significance of diastolic dysfunction signs [13-16]. Echocardiography remains the key modality in its estimation but is not without limitation. It is especially inconclusive for particular groups of patients. Present methods are not effective enough in determining diastolic dysfunction and assessing left ventricular filling pressure in HCM. Previous studies have shown that in contrast to other diseases, the mitral variables in the ratio ‘peak early filling to late diastolic filling velocities’ (E/A ratio) and deceleration time (DT), have weak to no correlation with the grade of diastolic dysfunction and left ventricle filling pressures in patients with HCM [17]. There are also discrepancies in the data about the application of the well-known ratio ‘peak mitral early diastolic filling velocity to early mitral annular velocity’ (E/e’ ratio) in the assessment of diastolic dysfunction and filling pressure [18-20].

In our study we selected the group of HCM patients which had no contraindications to exercise. Thereby all the subjects with an obstruction in the left ventricle outflow tract at rest were excluded. As a result our group came out as a sample of younger-aged patients with less manifestations of heart failure in comparison with the parent population. At rest this group had safe systolic and diastolic function according to conventional assessment. However, half of them had a history of dyspnea, a third of them presented a significant rise in the left ventricular outflow pressure gradient after stress, and an abnormal blood pressure response to exercise tests occurred in two patients. So abnormal tests were obtained in more than half of the cases. It is likely that these patients have some diastolic dysfunction especially during exertion, but present methods are not sensitive enough for detection in these cases.

In our present investigations we observed time parameters (TE, Te’, IVRT/(TE-Te’)) which were significantly different from controls and even from patients with arterial hypertension. Theoretically, these parameters can unmask impaired diastolic function in patients with HCM at rest and during exercise.

Relative time parameters TE/R-R and Te’/R-R likely reveal true changes in time parameters during exercise. Also an increase of Te’/R-R immediately after the stress indicates the delay of diastolic relaxation while decreasing IVRT or the absence of such significant changes of TE/R-R could be due to increasing pressure in the left atrium and a more rapid opening of the mitral valve. These changes were observed in HCM patients.

Previous investigations [18-20] have shown a bigger mean value of E/e’ in patients with HCM than was in our study. That can be explained by the different samples of subjects. The above-mentioned studies were confined to patients who were selected for operations. As the result, these people presented significant left ventricle outflow tract obstructions at rest, III-IV NYHA class of heart failure and more pronounced diastolic dysfunction.

### Study limitations

The patient selection was confined to those being in sinus rhythm, and HCM subjects being non-obstructive at rest.

We did not perform invasive measurements of the mean LV diastolic pressure for estimation of the mean left atrial pressure at rest and during exercise.

The number of patients in HCM group was limited for the purpose of dividing it into subgroups with and without abnormal test.

The group of AH was comparable with HCM group in their age and sex ratios so the result was also comprised of younger-aged patients with less degree of arterial hypertension.

The present data remains to be verified in future studies.
